# Isolation and Identification of a Strain of Aspergillus Tubingensis With Deoxynivalenol Biotransformation Capability

**DOI:** 10.3390/ijms9122366

**Published:** 2008-11-27

**Authors:** Chenghua He, Yanhong Fan, Guofang Liu, Haibin Zhang

**Affiliations:** College of Veterinary Medicine, Nanjing Agricultural University, Nanjing 210095, P.R. China. E-Mails: hechenghua2008@shou.com (C. H.); wittycat@sohu.com (Y. F.); mf_liuguofang@sina.com (G. L.)

**Keywords:** Trichothecene, Deoxynivalenol, Identification, Aspergillus tubingensis, Biotransformation

## Abstract

Deoxynivalenol (DON) is one of the most common contaminants of various foodstuffs. A biotransformation system was used in order to lessen the toxicity of DON. A strain of Aspergillus (*NJA-1*) was isolated from soil and cultured in an inorganic salt medium containing DON. Bt2a/Bt2b primers were used to amplify the *β-tubulin* gene of *NJA-1*. Sequence analysis the PCR product and morphology observation indicated that *NJA-1* belonged to *Aspergillus tubingensis* (aerobic fungi). The DNA sequence information of the PCR product was deposited in GenBank (accession number DQ9025790). The DNA sequence had 99% similarity to the *Aspergillus tubingensis* accession number AY820009. An unknown compound in *NJA-1* showed the ability to convert DON into another product. The molecular weight of the bioconversion product was 18.1 D (H2O) larger than that of DON. The analysis showed that DON could be hydrolyzed by *NJA-1*. The mean DON biotransformation rate was 94.4% after two weeks of cultivation. The finding presents a new method for DON biotransformation.

## 1. Introduction

Deoxynivalenol (DON), known as vomitoxin, a member of the trichothecenes which are secondary metabolites of fungi, mainly *Fusarium*, is one of the most common contaminants in various foodstuffs [1–5]. Epidemiological studies show an association of gastrointestinal diseases with DON, but critical data are still needed regarding its potential health damaging effects [6]. The toxic effects of DON in animals have been well documented and focused mainly on the immune system and the gastrointestinal tract [7]. In particular, the symptoms of acute toxicosis at high dosage include diarrhea, vomiting, leukocytosis, hemorrhage, circulatory shock and ultimately death [8]. Chronic toxicosis at low dosage leads to anorexia, reduced weight gain, nutrients malabsorption, neuroendocrine changes and immunosuppression [9–12]. Many methods are used for removal of mycotoxins in moldy feedstuff including chemical, physical and microbiological treatments [13–22]. However, because of DON’s relatively high chemical stability and nonpolar structure, physical treatments have a very low efficacy to remove DON. Meanwhile, some adsorbents can also absorb nutrients from the foodstuff, such as minerals and vitamins. Chemical treatment has a high efficacy on DON detoxification, but it can greatly reduce nutritive value and palatability of foodstuffs. Microbiological and enzymatic treatments are the best choice to reduce the toxicity of DON.

Some microbes have been reported to produce enzymes during growth which could lessen the toxicity of DON. The process has been also called “bio-transformation”. In present paper, a strain of *Aspergillus tubingensis* (*NJA-1*) was isolated from soil samples. The article mainly reports the isolation and identification of *NJA-1* and its efficacy on DON-transformation.

## 2. Results and Discussion

### 2.1. Isolation of NJA-1

Only one strain was isolated from fifteen soil samples and five rumen content samples and named *NJA-1*.

### 2.2. Identification of NJA-1

**Figure 1:**
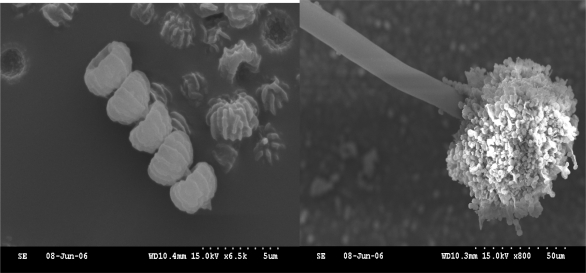
Electron microscope scanning photo of NJA-1. (A) spore; (B) sporangium.

The purified colonies had radial villus and white mycelia at the edge of each colony in early culture stages (3~5 d). After seven days, the colony became pitchy at the head of mycelium and white in the reverse side in Czapek’s Agar plate, respectively. The vesicle of *NJA-1* was round and radial. Diaphragms in hypha were visible with microscope. The value of metulae and conidial diameter were about 6 and 2.5 μm, respectively. Electron microscope scanning image showed that the spore was elliptical with introcession in head (Figure 1A). Sporangia of *NJA-1* consisted of many spores (Figure 1B).

**Figure 2:**
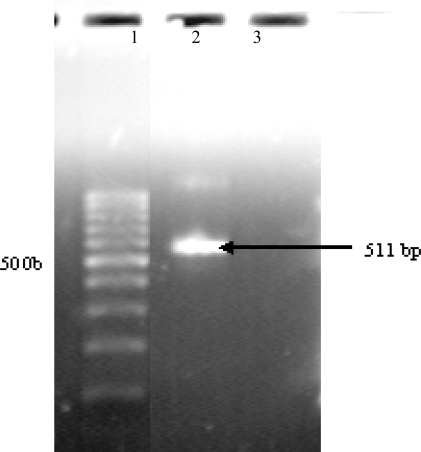
PCR amplification of *β-tubulin* gene. 1, DNA marker (100 bp); 2, PCR product of *β-tubulin* gene; 3, Water as template.

The *β-tubulin* gene of *NJA-1* was amplified with primers Bt2a–Bt2b. The size of amplified fragment was 511bp. Example of PCR result was shown in Figure 2. The results of the DNA sequence analysis were submitted to GenBank (access number DQ902579). The DNA sequence had 99% similarity to the *Aspergillus tubingensis* accession number AY820009. The results of the DNA sequence analysis and the observation of morphology showed that the *NJA-1* belonged to *Aspergillus tubingensis*.

### 2.3. The effect of transformation

**Figure 3:**
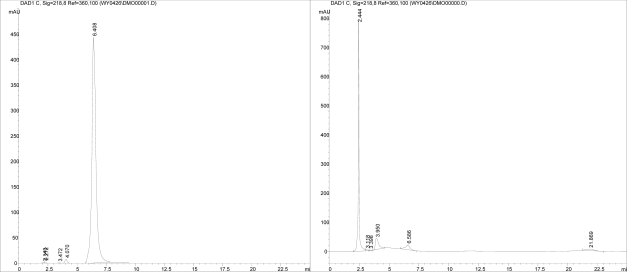
The detection of DON using HPLC (A: The peak time of DON was 6.408 min; B: The peak time of product was 2.444 min). X axis = time (min), Y axis = Absorbance.

The transformation results after 7d and 14d cultivation are shown in Table 1. The average transformation efficiency at 14 d was 94.4%. The detection of DON using HPLC is shown in Figure 3. The peak retention time of DON was 6.408 min, while that of the biotransformation product was 2.444 min. The difference between samples and control was significant (analyzed by SPASS 12.0 with One-Way ANOVA mode).

### 2.4. Screening for DON biotransformation product by NJA-1

**Figure 4:**
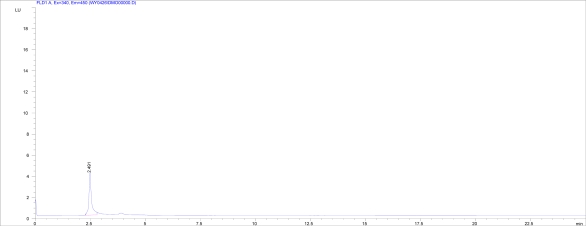
Fluorescence of the biotransformation product (Ex=340, Em=450) detected with HPLC equipped with fluorescence detector. The X axis = time (min), the Y axis = LU.

**Figure 5:**
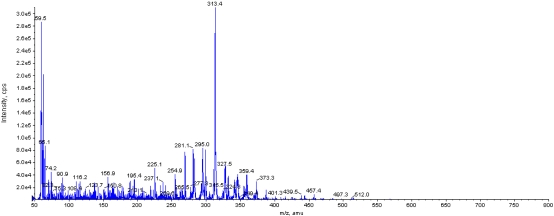
The detection of transformation product by HPLC-MS in negative ion mode (retention time 2.44 min, molecular weight 314.4). The X axis = z/m, the Y axis = intensity/cps.

The product of DON biotransformation by *NJA-1* was analyzed by HPLC and HPLC-MS. The HPLC diagram showed that the biotransformation product could emit fluorescence (Ex=340, Em=450) (Figure 4). The result of HPLC-MS showed that the molecular weight of biotransformation product was 314.4 (Figure 5).

Biotransformation of DON into less toxic metabolites by microorganisms was the most promising approach to counteract the mycotoxin. A bacterial strain BBSH 797 was isolated by Fuchs *et al*. [23]. The strain could transform DON into a lower toxicity metabolite DOM-1 (molecular weight 280.3). Strain E3-39 isolated from a soil sample was reported to convert DON to 3-keto-DON (molecular formula of C15H18O6) [24]. A mixed culture, D107, was reported to biologically degradate DON under anaerobic conditions [25]. A UDP-glycosyltransferase was able to lessen the toxicity of DON by transferring a glucose from UDP-glucose to the hydroxyl group at carbon 3 of DON [20]. In this study the strain of *Aspergillus tubingensis* (*NJA-1*) was isolated from soil by inorganic salt culture media supplemented with DON. DON was the only carbon source during the isolation. Because the growth of fungi required the carbon source and nitrogen source, the *NJA-1* could grow by using DON. This indicates that the *NJA-1* had the ability to transform the DON into another product. The hypothesis was confirmed by the HPLC and HPLC-MS results. The product is different from that of previous reports. The molecular weight of bioconversion product detected by HPLC-MS was 18.1 D (H2O) higher than that of DON, which would show that the DON could be hydrolyzed by *NJA-1*. The previous researches mainly focused on anaerobic bacteria. *NJA-1* was different from the most reported strains, because it is an aerobic fungi. We hope that the strain could have a wide application outlook for biotransformation of mycotoxin in mouldy feedstuffs, although further studies are needed to identify the biotransformation product of DON and compare the cytotoxicity of DON and its biotransformation product.

## 3. Experimental Section

### 3.1. Sampling

Fifteen soil samples (about 500 g per sample of clay soil was packed into sterile sampling bag and five rumen contents (about 10 mL per sample was extracted through stomach catheter and was placed in a 50 mL Erlenmeyer flask) were collected from Nanjing District and the dairy farm in Nanjing Agricultural University (NAU), respectively.

### 3.2. Culture media

Czapek’s Agar [26] contained (per liter): sucrose (30 g), NaNO_3_ (3 g), MgSO_4_·7H_2_O (0.5 g), KCl (0.5 g), FeSO_4_·7H_2_O (0.01 g), K_2_HPO_4_ (1 g), agar (13 g). The pH was adjusted to 7 with NaOH.

Inorganic salt culture media contained (per liter): (NH_4_)_2_SO_4_ (1.0 g), K_2_HPO_4_.3H_2_O (6.0 g), KH_2_PO_4_ (3.0 g), NaCl (0.5 g), MgSO_4_·7H_2_O (0.5 g), CaCl_2_ (0.05 g). The pH was adjusted to 7 with NaOH. Inorganic salt culture (50 mL) was added into a 250 mL conical flask. After autoclaving, DON (200 μg per conical flask, Sigma, purity: ≥97.0%) was added.

Liquid Czapek’s culture media was prepared similar to Czapek’s Agar but without the agar. Liquid Czapek’s culture media (50 mL) was added into a 250 mL conical flask. LB culture media contained (per liter): yeast extract (5 g), peptone (10 g), NaCl (10 g) and agar (15 g). The pH was adjusted to 7 with NaOH.

### 3.3. Purification and isolation

Soil (1 g) or rumen contents (1 mL) were added to sterile distilled water (50 mL), then mixed fully and centrifuged (2,000g × 10 min). Supernatant (1 mL) was added to inorganic salt culture media in a 250 mL conical flask in which DON was placed in advance. After cultivation at 30 °C for 7 d, a culture fluid sample (1 mL) was taken out and transferred into a 250 mL conical flask in which DON was added, and then was cultivated at 30 °C for another 7 d. Afterwards, a sample of the second culture fluid (1 mL) was spread on an inorganic salt culture media plate that contained DON. A single colony was cultured on Czapek’s Agar plate and LB culture media plate by streaking. A Strain of *Aspergillus Tubingensis* was isolated and named *NJA-1*.

### 3.4. Identification of NJA-1

#### 3.4.1. The observation of conventional morphology and scanning electron microscope

The taxonomic systems of Aspergillus were followed for observation and identification. The isolates were cultured on Czapek’s Agar at 30 °C. Macroscopical characters were assessed at were 3 and 7 days of age and microscopical characters were assessed after staining with lactophenol cotton blue. The colony color was observed by naked eye with the aid of color charts in the Methuen Handbook of Colour. The material on the slide mounts was examined under light microscope (OLYMPUS SZ-III Japan). The metulae, conidial diameter and conidial rough were determined under 1,000 × magnification. For scanning electron microscopic observations, the isolates were cultured on Czapek’s Agar at 30 °C for 7 days. The fungi were picked up on a 200-mesh copper screen and dried for viewing in the scanning electron microscope (HITACHI S-3000N Japan).

#### 3.4.2. Identification of *NJA-1* with PCR

The purified *NJA-1* was inoculated into liquid Czapek’s culture media and cultivated at 30 °C for 7 d, then thallus of *NJA-1*were collected by filtration. Extraction of DNA from *NJA-1* was performed following [27]. Almost the entire length of the *β-tubulin* gene of strain *NJA-1* was amplified by PCR with the primers Bt2a (5′-GGTAACCAAATCGGTGCTGCTTTC) and Bt2b (5′-ACCCTCAGTGTAGTGACCCTTGGC) [28]. Genomic DNA of *NJA-1* was added to PCR reactions containing forward and reverse primers. After 1 min incubation at 94 °C, thirty-three PCR cycles (94 °C, 1 min; 60 °C, 1 min and 72 °C, 1min) were performed, followed by one cycle of 10 min at 72 °C. The PCR product was separated by electrophoresis and then isolated and sequenced. A homology search was performed with GenBank database. The partial *β-tubulin* gene sequence was deposited into GeneBank Data Library with the accession number DQ902579.

### 3.5 The effect of DON transformation

The purified *NJA-1* was inoculated into inorganic salt culture media with DON at concentrations of 40, 2, 1 and 0.4 mg/L, respectively. At the same time, negative and positive controls were performed. Only *NJA-1* was added into the inorganic salt culture media as negative control and only DON was added as positive control. After cultivating at 30 °C for 14d, the concentration of DON was determined by high performance liquid chromatography (Hewlett Packard 1100 USA) equipped with fluorescence and UV detectors [29]. The HPLC conditions employed were as follows: Kromasil C_18_ column (250 × 4.6 mm, i.d., 5 μm); injection volume: 20 μL; column temperature: 29 °C; mobile phase: acetonitrile-water (15:85 v/v); flow rate: 0.8 mL/min; UV detection wavelength: 218 nm. To prevent trapping DON in cells and adsorption of DON to cell walls, cultures were split by ultrasonic wave before extraction. Three replicate experiments were run simultaneously. Data were analyzed by SPASS 12.0 with One-Way ANOVA mode.

### 3.6 Sscreening for DON biotransformation

An HPLC instrument (Hewlett Packard 1100 USA) equipped with UV-Vis diode array (G1315A) and fluorescence detectors (HP 1046A, USA) was used to screen the biotransformation product in culture media. The HPLC screening conditions were the same as the HPLC conditions for determination, except the UV-Vis diode array detector wavelength of 190–630 nm setting and fluorescence detector operated at Ex = 340, Em = 450.

An HPLC-MS instrument (Hewlett Packard 1100/API 2000) with atmospheric pressure chemical ionization (APCI) in negative ion mode were used to screen the DON product. The MS conditions employed were as follows: ion source temperature: 200 °C; APCI detection temperature: 450 °C; scanning range: m/z 50–800.

## 4. Conclusions

A strain of Aspergillus (*NJA-1*) was isolated from soil by inorganic salt culture media supplemented with DON. Sequence analysis of *Bt2a–Bt2b gene* and morphology observation indicated that *NJA-1* belong to *Aspergillus tubingensis* (aerobic fungi). The DNA sequence of *Bt2a–Bt2b* PCR product was deposited in GenBank (accession number DQ9025790). The mean DON biotransformation rate was 94.4% after two weeks cultivation. The product of DON transformed by *NJA-1* could emit fluorescence, and its molecular weight was 18.1 D (H_2_O) larger than that of DON analyzed by HPLC-MS. The analysis suggested that DON could be hydrolyzed by *NJA-1*. The finding could present a new method for DON biotransformation. To authors’ knowledge, it is the first report that *Aspergillus tubingensis* has the ability for DON biotransformation.

## Figures and Tables

**Table 1:** The concentration of DON in culture after cultivation for 7 d and 14 d.

No. of sample	Original concentration (mg/L)	Concentration after 7d cultivation. (mg/L)	Concentration after 14d cultivation. (mg/L)	Transformation efficiency at 14d (%)
1	40	20.32±0.26	3.28±0.02	91.8%^*^
2	2	1.2±0.06	0.139±0.02	93.1%^*^
3	1	0.56±0.16	0.035±0.05	96.5%^*^
4	0.4	0.18±0.056	0.015±0.07	96.2%^*^
negative control	0	0	0	0
positive control	0.5	0.498±0.003	0.497±0.01	0.6%

Note: * indicates significant difference between samples and positive control (P <0.01).
